# Comparative Transcriptome Analysis of the Hepatopancreas from *Macrobrachium rosenbergii* Exposed to the Heavy Metal Copper

**DOI:** 10.3390/ani14071117

**Published:** 2024-04-05

**Authors:** Jiayuan Zhang, Zhiming Bao, Jieyu Guo, Xianbin Su, Yongfeng Zou, Hui Guo

**Affiliations:** 1 College of Fisheries, Guangdong Ocean University, Zhanjiang 524025, China; jiayuan1629@163.com (J.Z.); baozhiming@stu.gdou.edu.cn (Z.B.); guojieyu888@gmail.com (J.G.); 2112201127@stu.gdou.edu.cn (X.S.); zouyf111@126.com (Y.Z.); 2 Guangdong Provincial Key Laboratory of Aquatic Animal Disease Control and Healthy Culture, Zhanjiang 524088, China

**Keywords:** transcriptome, *Macrobrachium rosenbergii*, hepatopancreas, copper exposure, molecular mechanism

## Abstract

**Simple Summary:**

The pollution of aquatic ecosystems with the heavy metal copper (Cu) presents considerable risks to the physiological functions of aquatic organisms, including the economically valuable *Macrobrachium rosenbergii*. In this study, transcriptomic analysis was conducted to explore the molecular response mechanisms in the hepatopancreas of *M. rosenbergii* exposed to Cu. The results showed that *M. rosenbergii* attempts to resist the toxicity of Cu by up-regulating the expression of genes related to immunity, metabolism, and detoxification. However, with the excessive accumulation of reactive oxygen species (ROS), the antioxidant enzyme system was destroyed. As a result, DNA damage repair and the cellular stress response were inhibited, thereby exacerbating cell damage. In order to maintain the normal function of the hepatopancreas, *M. rosenbergii* removes damaged cells by activating the apoptosis mechanism. Differentially expressed genes (DEGs) and KEGG pathways identified in the present study not only facilitate an understanding of the molecular response mechanisms of *M. rosenbergii* underlying Cu toxicity effects but also help in identifying potential biomarkers associated with the stress response in other crustaceans.

**Abstract:**

The contamination of aquatic ecosystems by the heavy metal copper (Cu) is an important environmental issue and poses significant risks to the physiological functions of aquatic organisms. *Macrobrachium rosenbergii* is one of the most important freshwater-cultured prawns in the world. The hepatopancreas of crustaceans is a key organ for immune defense, heavy metal accumulation, and detoxification, playing a pivotal role in toxicological research. However, research on the molecular response of the hepatopancreas in *M. rosenbergii* to Cu exposure is still lacking. In this study, the transcriptomic response in the hepatopancreas of *M. rosenbergii* was studied after Cu exposure for 3 and 48 h. Compared with the control group, 11,164 (7288 up-regulated and 3876 down-regulated genes) and 10,937 (6630 up-regulated and 4307 down-regulated genes) differentially expressed genes (DEGs) were identified after 3 and 48 h exposure, respectively. Most of these DEGs were up-regulated, implying that gene expressions were largely induced by Cu. Functional enrichment analysis of these DEGs revealed that immunity, copper homeostasis, detoxification, DNA damage repair, and apoptosis were differentially regulated by Cu. Seven genes involved in immunity, detoxification, and metabolism were selected for validation by qRT-PCR, and the results confirmed the reliability of RNA-Seq. All these findings suggest that *M. rosenbergii* attempts to resist the toxicity of Cu by up-regulating the expression of genes related to immunity, metabolism, and detoxification. However, with the excessive accumulation of reactive oxygen species (ROS), the antioxidant enzyme system was destroyed. As a result, DNA damage repair and the cellular stress response were inhibited, thereby exacerbating cell damage. In order to maintain the normal function of the hepatopancreas, *M. rosenbergii* removes damaged cells by activating the apoptosis mechanism. Our study not only facilitates an understanding of the molecular response mechanisms of *M. rosenbergii* underlying Cu toxicity effects but also helps us to identify potential biomarkers associated with the stress response in other crustaceans.

## 1. Introduction

Copper (Cu), a redox-active transition metal, is a double-edged sword. On the one hand, Cu is an indispensable micronutrient for the development and homeostasis of all living organisms [1]. For example, it is an important cofactor of enzymes, including copper–zinc superoxide dismutase (SOD 1), cytochrome c oxidase (CCO), and lysyl oxidase (LOX) [2]. On the other hand, excessive Cu in the aquatic environment leads to the accumulation of reactive oxygen species (ROS) in aquatic organisms, leading to DNA damage, apoptosis, and tissue structure damage [3,4,5]. In addition, Cu can cause damage to the mitochondrial membrane of crustaceans and disrupt normal metabolism [6]. In recent years, Cu has been widely used in industrial production activities as an important conductive material and raw material for manufacturing alloys, as an effective algaecide for inhibiting toxic algal blooms, and as an essential mineral in feed [7,8,9,10]. These activities undoubtedly cause Cu contamination in aquaculture environments. The latest research showed that Cu pollution in the lower reaches of the Yangtze River is already widespread [11].

*Macrobrachium rosenbergii* is primarily distributed in inland freshwater areas. Because of its short culture cycle, high nutritional value, and other advantages, it has become an important economic species in China [12]. In addition, *M. rosenbergii* has always been considered an important model organism with relevance for many research fields such as ecology [13], physiology [14], and toxicology [15]. The hepatopancreas has metabolic, immunological, and antidotal functions, which are important for the healthy growth of crustaceans and resistance to external environmental stimuli [16,17]. Numerous studies have found that the hepatopancreas is a key target organ for heavy metal accumulation [18,19]. High concentrations of heavy metals in aquatic environments can lead to tissue damage, metabolic disorders, and immune suppression in the hepatopancreas [20,21,22]. Up to now, the effects of Cu exposure on the hepatopancreas of *M. rosenbergii* have mainly focused on tissue enrichment [23], structural damage [24], and enzyme activity [25], while the specific molecular response mechanisms of the hepatopancreas remain poorly understood.

Transcriptome sequencing could identify genes and pathways involved in immunity and metabolism under environmental pollution stress, and it has been widely used in the study of the stress response of aquatic animals [26,27]. In this study, transcriptome sequencing was used to obtain genes and pathways that respond to Cu exposure in the hepatopancreas of *M. rosenbergii* at different time points, and to explore the molecular mechanisms against Cu toxicity. These findings not only provide new insights into the molecular response mechanisms underlying Cu toxicity effects but also help in identifying potential biomarkers associated with the stress response in other crustaceans.

## 2. Materials and Methods

### 2.1. Experimental Animal

The experimental *M. rosenbergii* (10.89 ± 1.42 g, both genders) were raised at the Donghai Island Research Base of Guangdong Ocean University. *M. rosenbergii* were acclimatized in three cycling-filtered water tanks containing aerated and filtered freshwater (200 L) for 2 weeks. The water temperature, pH, and dissolved oxygen (DO) were 24–28 °C, 7.5–7.9, and 5.36–6.18 mg/L, respectively. Half of the water was renewed daily to maintain good water quality. During this period, the prawns were fed twice a day with a commercial diet (supplied by Haid Group Co., Ltd., Zhanjiang, China) until 24 h before the beginning of the Cu exposure experiment.

### 2.2. Cu Exposure Experiment and Sample Collection

In order to investigate the physiological response of *M. rosenbergii* to Cu exposure, these *M. rosenbergii* were randomly divided into two groups. The control group was still in the initial conditions, and the exposed group was conducted in the tank containing 200 L of Cu solution (100 μg/L) [28]. Each group was conducted in three replicates, with twenty prawns per tank. The desired Cu concentration was attained by adding CuSO_4_ to freshwater. The Agilent model 7500cx ICP-MS (Agilent Technologies, Santa Clara, CA, USA) detection system was used to determine the actual Cu concentrations, using operating parameters recommended by the manufacturer. The actual Cu concentrations of the experiment were 11.0 (control) and 98.9 μg/L (mean, n = 3), respectively. At the beginning of the experiment, nine samples were randomly selected from the control group (Hep_0 h) to collect their hepatopancreases. Nine hepatopancreas samples were collected from the exposed group at 3 h (Hep_3 h) and 48 h (Hep_48 h), respectively. All of the hepatopancreas tissues were immediately placed in liquid nitrogen for freezing and then stored at −80 °C until RNA extraction.

### 2.3. RNA Extraction, Library Construction, and RNA Sequencing

Total RNA was extracted from the hepatopancreas of *M. rosenbergii* using Trizol reagent (Invitrogen, Carlsbad, CA, USA) according to the manufacturer’s protocol. The quantity and quality of these RNA samples were detected by measuring their absorbance at 260 and 280 nm using a NanoDrop 2000 spectrophotometer (NanoDrop Technologies, Wilmington, DE, USA). The integrity of RNA was assayed by agarose gel electrophoresis. After qualification, equal amounts of RNA from nine individuals in each group were mixed together as a sample to construct the transcriptome library according to the instructions of the Illumina TruSeq RNA Sample Preparation Kit (Illumina Inc., San Diego, CA, USA). In brief, after treating total RNA with DNasel, mRNA was enriched using oligo (dT) magnetic beads. Then, fragmentation buffer was added to break the purified mRNA into short fragments (about 200 bp). These small mRNA fragments were used as templates to synthesize the first complementary DNA (cDNA) strand using random hexamers, Rnase H and SuperScript II Reverse Transcriptase (Invitrogen, Life Technologies). Next, the second strand of cDNA was synthesized, followed by adhesive end repair and the addition of base A at 3′ ends. Proper cDNA fragments were purified by agarose gel electrophoresis and subsequently enriched by PCR amplification. After passing the quality inspection of the Agilent 2100 Bioanalyzer (Agilent Technologies Inc., Santa Clara, CA, USA) and the ABI StepOnePlus Real-Time PCR System (Thermo Fisher Scientific, MA, USA), the constructed libraries were sequenced using the Illumina Hiseq 4000 platform, and 150 bp paired-end raw reads were generated.

### 2.4. Transcriptome Assembly and Functional Annotation Analysis

Adaptor sequences, sequences with more than 5% N bases, and low-quality sequences (more than 50% bases with quality value ≤ 10) were removed from the raw reads. De novo assembly of the remaining clean reads was performed using Trinity software (version: trinityrnaseq_r20140717) to generate large contigs [29]. TGICL V2.1 was used to eliminate redundant sequences and perform additional assembly, resulting in transcripts called unigenes [30]. All unigenes were annotated by comparison with non-redundant protein (Nr), non-redundant nucleotide (Nt), SwissProt, Gene Ontology (GO), Clusters of Orthologous Groups of Proteins (COG), and Kyoto Encyclopedia of Genes and Genomes (KEGG) databases using BLAST (version: v2.2.26), with a cut-off *E*-value of 1 × 10^−5^ [31]. Based on the Nr BLAST results, Blast2GO version 2.6.6 was used to conduct GO annotation [32]. KEGG pathway analysis was conducted using KOBAS standalone version 2.0 [33].

### 2.5. Identification of Differentially Expressed Genes (DEGs) and Functional Enrichment Analysis

The expression level of each unigene was calculated and standardized by Fragment Per Kilo bases per Million Reads (FPKM), using the software RNA-Seq by Expectation Maximization (RSEM: v1.2.21) [34]. Differentially expressed genes (DEGs) were identified by the R package DEGseq (version: v1.18.0) [35] with the threshold set to |Log2Fold Change| ≥ 1 and a false discovery rate (FDR) ≤ 0.01 [36]. Then, all DEGs in the three paired comparisons (Hep_0 h vs. Hep_3 h, Hep_0 h vs. Hep_48 h, and Hep_3 h vs. Hep_48 h) were subjected to GO functional and KEGG pathway enrichment analysis using Goseq V1.16.2 [37] and KOBAS [33], respectively. We defined the *p*-value < 0.05 as significantly enriched.

### 2.6. Simple Sequence Repeat (SSR) and Single-Nucleotide Polymorphism (SNP) Detection

Simple sequence repeats (SSRs) were identified by using MISA version 2.2.2 [38]. Specific selection criteria were as follows: mono-nucleotide ≥ 12, di-nucleotide ≥ 6, tri-nucleotide and tetra-nucleotide ≥ 5, penta- and hexa-nucleotide ≥ 4. PCR primers for each detected SSR were designed using Primer3 with default parameters. A single-nucleotide polymorphism (SNP) primarily refers to the polymorphism of a DNA sequence caused by the variation of a single nucleotide at the genomic level. We employed the Genome Analysis Toolkit (GATK, version 3.4.0) for the detection of SNP markers [39]. Subsequently, the generated raw Variant Call Format (VCF) files were subjected to filtration using the GATK’s prescribed standard filtering methodology.

### 2.7. Validation of RNA-Seq Results with Quantitative Real-Time PCR (qRT-PCR)

Seven DEGs involved in immunity, detoxification, and metabolism, such as *relish*, glucose transporter 2 (*Glut 2*), JHE-like carboxylesterase 2 (*CXE2*), hemocyanin (*HC*), glutathione S-transferase (*GST*), serine/threonine–protein phosphatase 6 regulatory subunit 3 (*SAPS3*), and C-type lectin (*CTL*), were selected to validate the RNA-Seq results, and the elongation factor-1 gene (*EF-1*) was chosen as an internal reference gene [40]. These genes were analyzed by qRT-PCR. Primers for qRT-PCR were designed by Primer Premier 6.0 (Premier Biosoft, Palo Alto, CA, USA), as shown in Table 1. qRT-PCR was conducted with ChamQ Universal SYBR qPCR Master Mix (Vazyme, Nanjing, China), with the following conditions: 95 °C for 6 min, followed by 40 cycles of 95 °C for 5 s, 60 °C for 15 s, 72 °C for 10 s. The relative gene expression levels of the chosen genes were calculated using the 2^−ΔΔCt^ method [41].

## 3. Results

### 3.1. RNA Sequencing and De Novo Assembly

The transcriptome sequencing results are shown in Table 2. A total of 179,549,702 raw reads were generated from the three cDNA libraries of *M. rosenbergii*. After removing the reads containing adapter sequences and low-quality sequences, a total of 178,793,124 clean reads were obtained. The Trinity software (version: trinityrnaseq_r20140717) was used to assemble the obtained clean reads, and a total of 85,984 unigenes were generated (with a mean length of 1077.23 bp and N50 of 2559 bp) (Table S1). A total of 27,679 unigenes at 200–300 bp accounted for the largest ratio, as high as 32.19%. There were only 8010 unigenes with a length greater than 3000 bp, accounting for 9.32% of the total (Figure S1).

### 3.2. Functional Annotation and Classification of the Transcriptome

Among all the assembled sequences, a total of 85,984 unigenes were annotated within six functional databases (Table S2), and it was found that 10,741 (12.49%), 13,535 (15.74%), 20,601 (23.96%), 24,394 (28.37%), 13,110 (15.25%), and 20,528 (23.87%) unigenes could find significant hits in the COG, GO, KEGG, Nr, Nt, and SwissProt databases, respectively (Figure S2). A total of 5614 unigenes (6.53%) were mapped to all databases (Figure S3). The species distribution of the most significant hits in the Nr database was analyzed, and the results showed that the maximum number of homologous sequences was found in the arthropods *Zootermopsis nevadensis* and *Limulus polyphemus* with high percentages of 10.59% (2583) and 5.55% (1353), respectively. The percentage of unigenes matched to several crustacean species was relatively low, such as in *Litopenaeus vannamei* (1.29%), *Penaeus monodon* (0.93%), *Marsupenaeus japonicus* (0.74%), *Macrobrachium nipponense* (0.73%), *Procambarus clarkii* (0.68%), and *M. rosenbergii* (0.68%) (Figure S4), which may be due to the lack of gene/protein information on crustaceans in the database.

The GO functional analysis showed that a total of 13,535 unigenes were arranged into three main categories: biological process, cellular component, and molecular function. In the biological process category, a large percentage of the unigenes belonged to cellular process (4923), followed by metabolic process (4848) and single-organism process (3746). In the cellular component category, the dominant subcategories were ordered as follows: cell (2100), cell part (2100), membrane (1943). In the molecular function category, most unigenes were related to binding (8403), catalytic activity (4478), and transporter activity (699) (Figure S5). KEGG functional analysis indicated that a total of 20,601 unigenes were mapped to six major types of pathways, namely genetic information processing, metabolism, cellular processes, organismal systems, human diseases, and environmental information processing. The top three pathways with significant unigenes were signal transduction, global and overview maps, and endocrine system. In addition, the immune system pathway was also significantly enriched (Figure S6).

### 3.3. Identification of Differentially Expressed Genes (DEGs)

**Figure 1:**
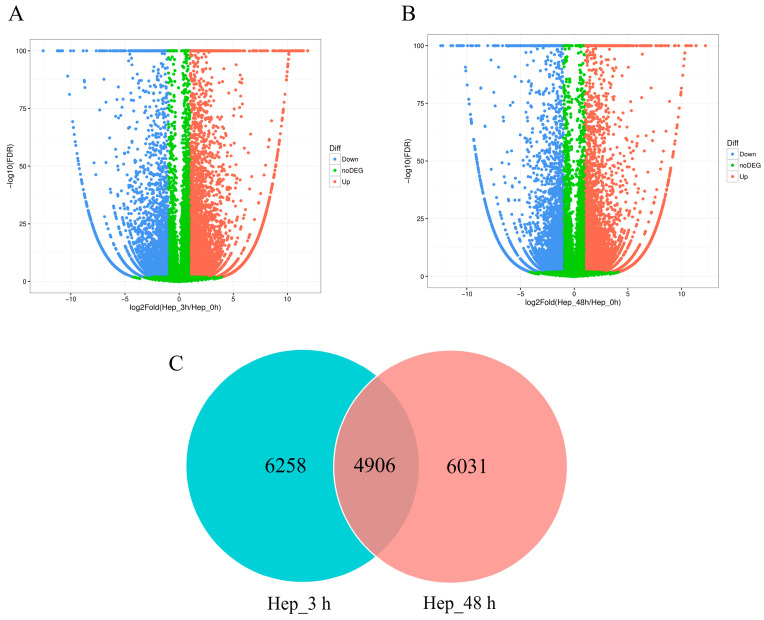
(**A**) Volcano Plot of DEG distribution trends in the Hep_3 h vs. Hep_0 h comparison. Each dot represents one gene. Red dots represent up-regulated genes and blue dots represent down-regulated genes. Green dots represent no DEGs. (**B**) Volcano Plot of the distribution trends of DEGs in the Hep_48 h vs. Hep_0 h comparison. (**C**) Venn diagrams show the DEGs in the Hep_3 h vs. Hep_48 h comparison.

Gene expression changes of *M. rosenbergii* at different treatment time points after Cu exposure were analyzed by pairwise comparison (Table S3). The statistical results showed that, compared with the control group, 11,164 (7288 up-regulated and 3876 down-regulated genes) and 10,937 (6630 up-regulated and 4307 down-regulated genes) DEGs were screened out after 3 h and 48 h exposure, respectively (Figure 1A,B). With regard to the comparison between 3 and 48 h, a total of 6224 down-regulated and 5274 up-regulated genes were identified (Figure S7). Among these DEGs, 4906 genes were both changed at 3 and 48 h, while 6258 genes exhibited differential expression just at 3 h, and 6031 genes exhibited differential expression just at 48 h (Figure 1C). These time-specific genes may be helpful in clarifying stress response indicators at different time points. 

### 3.4. GO and KEGG Enrichment Analyses of DEGs

**Figure 2:**
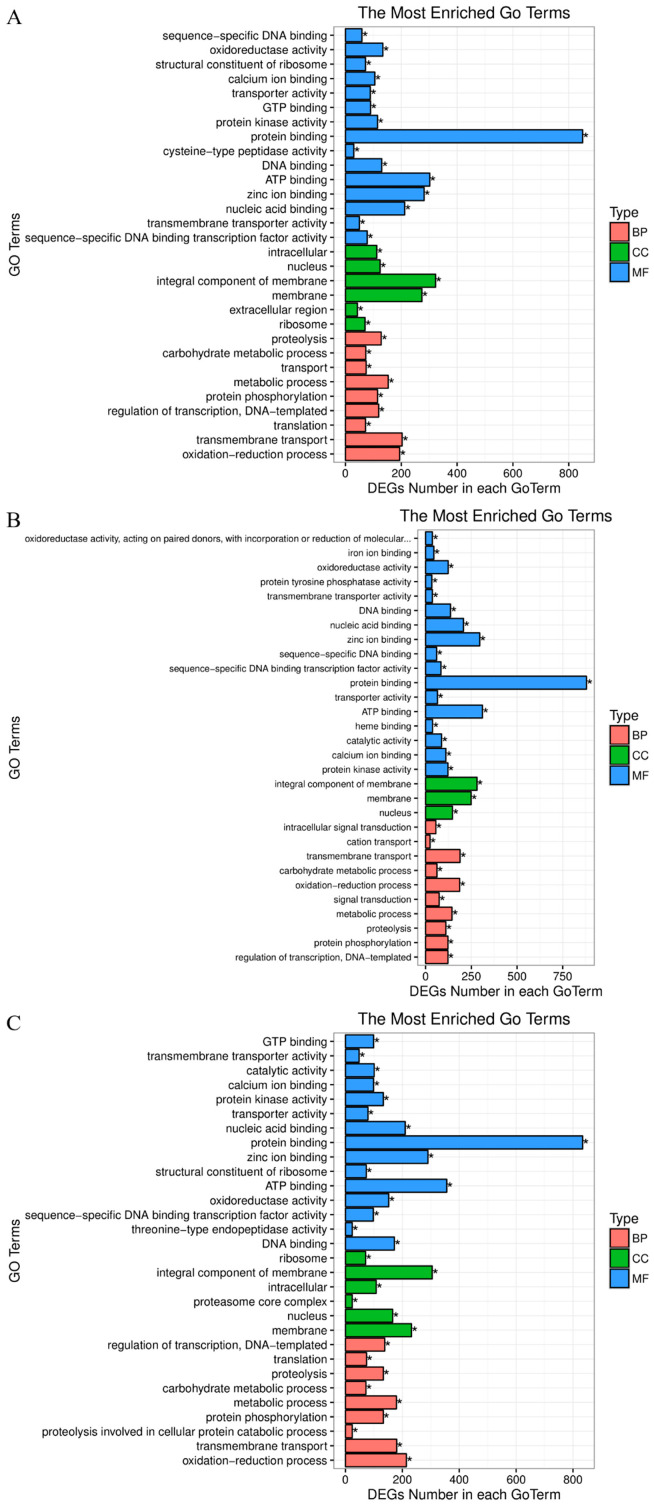
Gene Ontology assignments of DEGs in the (**A**) Hep_0 h vs. Hep_3 h, (**B**) Hep_0 h vs. Hep_48 h, and (**C**) Hep_3 h vs. Hep_48 h comparisons. (* means correct *p*-value < 0.05).

In order to identify the biological processes and pathways of DEGs that may be involved in the Cu stress response, GO and KEGG enrichment analyses of all DEGs of the three comparisons (Hep_0 h vs. Hep_3 h, Hep_0 h vs. Hep_48 h, and Hep_3 h vs. Hep_48 h) were conducted. The GO functional enrichment analysis showed that DEGs were classified into three categories: molecular function, biological process, and cell component. The top 30 GO terms enriched in each group are shown in Figure 2. In the molecular function and cellular component categories, the subcategories with the most DEG enrichment among the three comparisons were protein binding (GO:0005515) and integral component of membrane (GO:0016021), respectively. When it comes to biological processes, the most DEGs were enriched in the transmembrane transport subcategory (GO:0055085) in the Hep_0 h vs. Hep_3 h and Hep_0 h vs. Hep_48 h comparisons, while most DEGs were mainly enriched in the oxidation–reduction process subcategory (GO:0055114) in the Hep_3 h vs. Hep_48 h comparison. 

**Figure 3:**
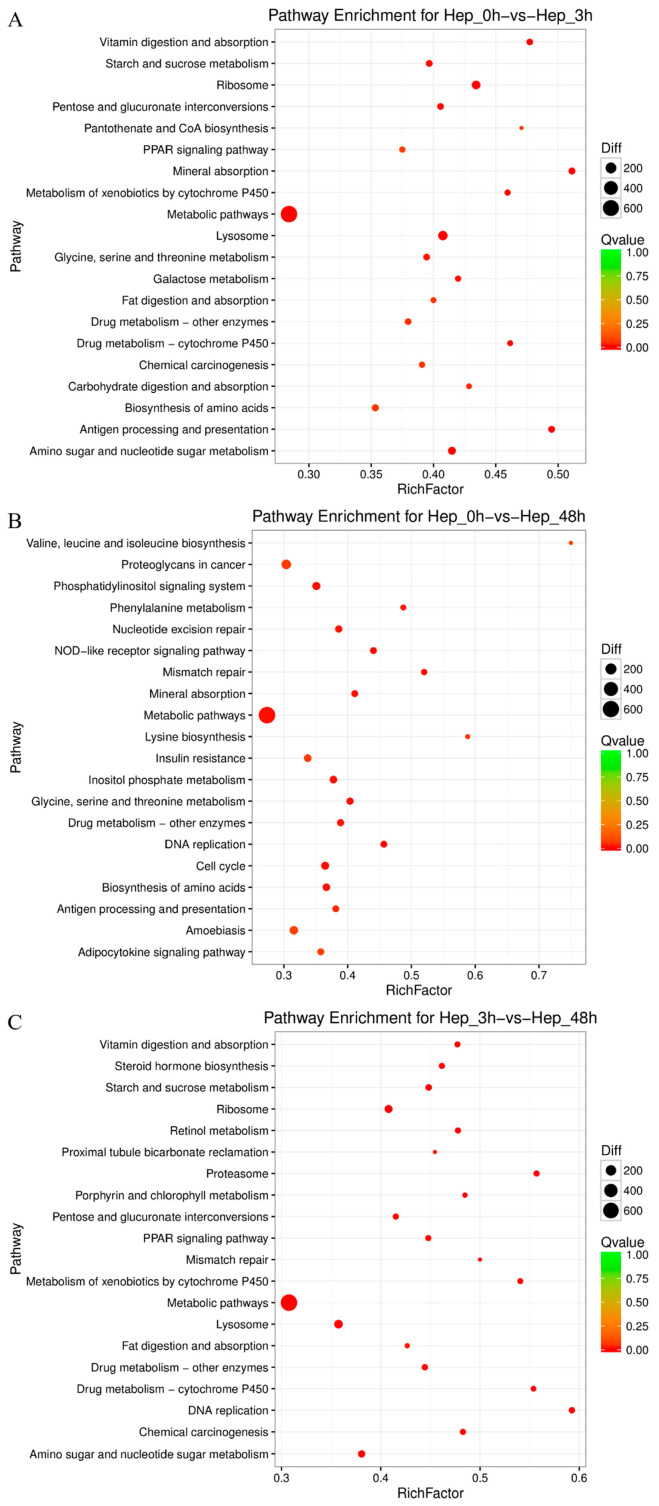
Comparative KEGG pathways analysis in the (**A**) Hep_0 h vs. Hep_3 h, (**B**) Hep_0 h vs. Hep_48 h, and (**C**) Hep_3 h vs. Hep_48 h comparisons.

To better understand the functional characteristics of these DEGs, the KEGG pathway functional enrichment of DEGs was performed. A total of 299, 298, and 297 pathways were annotated, and 49, 53, and 55 pathways were significantly enriched in Hep_0 h vs. Hep_3 h, Hep_0 h vs. Hep_48 h, and Hep_3 h vs. Hep_48 h, respectively (*p*-value < 0.05). The top 20 KEGG enrichment analyses of DEGs for each comparison are shown in Figure 3. Of them, multiple pathways are associated with immunity, for example, “Lysosome” (ko04142) and “Metabolism of xenobiotics by cytochrome P450” (ko00980) in Hep_0 h vs. Hep_3 h, “NOD-like receptor signaling pathway” (ko04621) and “Amoebiasis” (ko05146) in Hep_0 h vs. Hep_48 h, and “Proteasome” (ko03050) in Hep_3 h vs. Hep_48 h. It also provided some other molecular pathways related to metabolism and detoxification, such as “Glycine, serine and threonine metabolism” (ko00260), “Metabolic pathways” (ko01100), “Drug metabolism-other enzymes” (ko00983), “Drug metabolism-cytochrome P450” (ko00982), etc. In addition, it is interesting to note that pathways such as “Cell cycle” (ko04110), “Mineral absorption” (ko04978), “Mismatch repair” (ko03430), “Nucleotide excision repair” (ko03420), and “Base excision repair” (ko03410) were also affected after Cu exposure. Taken together, these results indicated that genes related to oxidation–reduction, energy metabolism, apoptosis, copper homeostasis, detoxification, immune response, and DNA damage repair in the hepatopancreas of *M. rosenbergii* were changed after Cu exposure (Table 3).

### 3.5. Detection of SSR and SNP Loci in the Transcriptome

**Figure 4:**
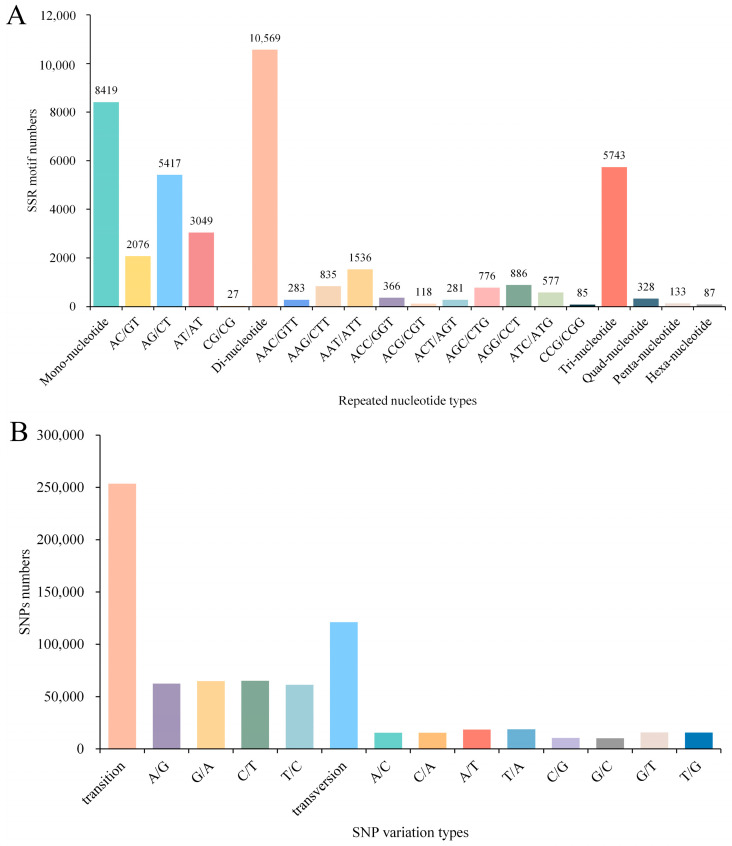
Distribution of identified SSRs (**A**) and putative SNPs (**B**) in the transcriptome.

A total of 25,279 SSRs were identified across 85,984 unigenes, with the majority of SSR repeats being di-nucleotide repeats (10,569, 41.81%), followed by mono-nucleotide repeats (8419, 33.30%) and tri-nucleotide repeats (574,379, 22.72%), and the rest being quad-nucleotide repeats (328, 1.30%), penta-nucleotide repeats (133, 0.53%), and hexa-nucleotide repeats (87, 0.3%). Among the di-nucleotide repeats, AG/CT was the main type, followed by AT/AT and AC/GT. The most common repeat sequences in tri-nucleotide repeats were AAT/ATT and AGG/CCT (Figure 4A). Detailed SSR results and corresponding primers are presented in Table S4. In addition, a total of 374,631 SNPs were predicted, including 253,540 transition and 121,091 transversion, with C/T (65,001) and T/A (18,811) SNP types being the most frequent in transition and transversion, respectively (Figure 4B).

### 3.6. The qRT-PCR Validation of Differentially Expressed Genes

**Figure 5:**
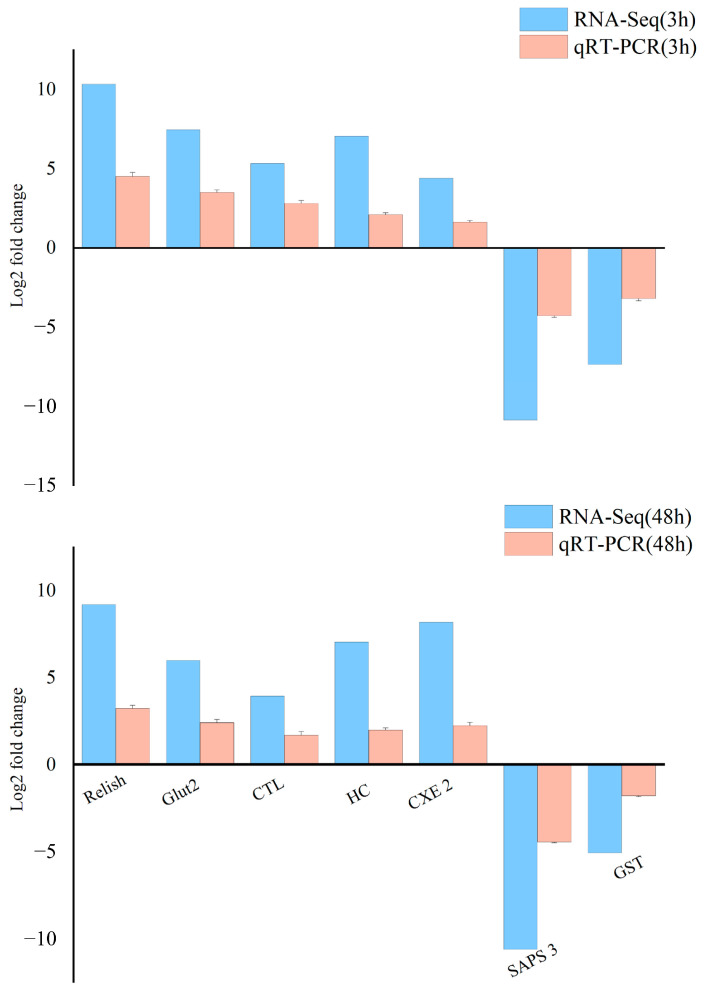
Comparison of gene expression data between RNA-Seq and qRT-PCR.

Seven DEGs were chosen for validation by qRT-PCR. The results showed that, compared to the control group, the expression levels of *relish*, *Glut 2*, *HC*, *CXE 2*, and *CTL* were up-regulated, while *SAPS 3* and *GST* were down-regulated. These expression trends were consistent with those observed in the high-throughput sequencing data (Figure 5), confirming the precision and reliability of the sequencing results.

## 4. Discussion

Cu, a common heavy metal contaminant in aquatic ecosystems, has raised considerable global concern [11]. Currently, the detailed molecular response mechanisms in the hepatopancreas of *M. rosenbergii* under Cu exposure remain poorly elucidated. In this study, RNA-Seq technology was utilized to reveal the molecular response mechanisms of the hepatopancreas of *M. rosenbergii* after Cu exposure for 3 and 48 h. Our research was designed to compare the temporal dynamics of stress responses in the hepatopancreas of *M. rosenbergii*, with the aim of identifying DEGs and disparate pathways involved. A total of 85,984 unigenes were de novo assembled, yet only 28,668 (33.34%) were annotated. The limited number of annotated unigenes may reflect the scarcity of available reference sequences for crustaceans and related species in public databases. Upon Nr database annotation, the largest number of the annotated unigenes were most closely related to *Z. nevadensis* and *L. polyphemus*. Notably, a substantial portion remained unannotated; this result was consistent with a previous transcriptomic analysis of *M. rosenbergii* [42]. The unannotated unigenes might serve as prospective candidates for future explorations in gene discovery.

A total of 11,164 (7288 up-regulated and 3876 down-regulated genes) and 10,937 (6630 up-regulated and 4307 down-regulated genes) DEGs were discovered at 3 and 48 h, respectively. We observed that the number of up-regulated genes was much greater than the down-regulated genes at both time points. Similar results were also reported in previous studies on *Neocaridina denticulate sinensis* and *Anodonta woodiana*, indicating that Cu might induce the expression of a greater number of genes in response to Cu toxicity [4,43]. Subsequently, GO and KEGG pathway enrichment analysis of these DEGs revealed that these DEGs play a pivotal role in a suite of fundamental biological processes, such as oxidation–reduction, copper homeostasis, energy metabolism, apoptosis, detoxification, immune response, and DNA damage repair. The temporal specificity of these genes not only highlights the dynamic nature of the stress response but also provides new insights into the time-regulated molecular mechanisms during Cu exposure.

Similar to other invertebrates, *M. rosenbergii* rely on innate immunity as their primary defense mechanism against external environmental stressors. The Toll-like receptor (TLR) was the first identified pattern recognition receptor (PRR), and its role in innate immunity has been characterized in several crustacean species [44,45,46]. A previous report observed that the expression of *TLR* in the hepatopancreas was induced by heavy metal stimulation in a time-dependent manner [47]. CTL and ficolin-like protein 2 (FLP 2) are also integral components of the PRRs [48,49]. For crustaceans, CTL and FLP 2 play crucial roles in immune defense, immunosurveillance, and the maintenance of body homeostasis [49,50]. In our transcriptome results, *TLR* (CL4523.Contig2_All), *CTL* (CL3394.Contig2_All), and *FLP 2* (CL4909.Contig1_All) were up-regulated, implying that *M. rosenbergii* may mitigate the adverse effects of Cu exposure by enhancing the binding activity of PRRs, thereby bolstering its immune defense mechanisms. Antimicrobial peptides (AMPs) constitute the initial line of defense in the host’s innate immune system. It has been found that *domeless*, *stat*, and *relish* are all involved in the regulation of AMP expression at various levels in *P. clarkii*, *M. rosenbergii*, and *Eriocheir sinensis* [51,52,53]. Hemocyanin (HC), a precursor to AMPs, not only serves as an oxygen carrier but also plays a role in immune defense [54,55,56]. The expression level of *HC* was up-regulated in crustaceans following heavy metal exposure, thereby facilitating an immunological response [10,42]. In our study, *HC* (CL2.Contig14_All), *domeless* (CL5288.Contig1_All), *stat* (CL607.Contig1_All), and *relish* (CL2513.Contig3_All) were significantly induced after Cu exposure, indicating that the up-regulation of these genes might be a critical response in the resistance of *M. rosenbergii* to Cu toxicity. The above findings indicated that *M. rosenbergii* may activate a diverse array of immune mechanisms to counteract the physiological challenges induced by Cu exposure.

In crustaceans, the hepatopancreas is the target organ for the accumulation of heavy metals as well as a vital detoxification organ. The high-affinity copper uptake protein 1 (Ctr1) is primarily responsible for the absorption of Cu [57]. Excessive Cu decreased the expression of *Ctr1*, preventing the intracellular accumulation of Cu [58]. In contrast, in our study, Cu induced the expression of *Ctr1* (CL2846.Contig2_All). This might lead to an excessive influx of Cu into the cells, resulting in toxicity [59]. The occurrence of this intriguing phenomenon may be due to interspecific differences in the response of organisms to Cu exposure. Metallothioneins (MTs, Cu-MT, and Zn-MT) are the main metal-binding thiol proteins, protecting aquatic organisms against the toxic effects of heavy metals through the sequestration and regulation of essential metal ions [60]. Cytochrome c oxidase Cu chaperone (COX17) is a Cu-chelating protein responsible for the intracellular transport of Cu ions to mitochondria, participating in the enzymatic reactions mediated by cytochrome c oxidase [61]. ATP7A participates in the elimination of excess Cu from cells to regulate the intracellular concentration of Cu [62]. In this study, significant up-regulation of *COX17* (Unigene9795_All), *Cu-MT* (Unigene13182_All), and *ATP7A* (CL3799.Contig3_All) was observed. We assumed that *M. rosenbergii* may mitigate the toxicity of intracellular Cu accumulation by sequestering, utilizing, and excreting excess Cu. In addition, JHE-like carboxylesterase 2 (CXE2) and cytochrome P 450 (CYP 450) are involved in detoxification and protection against oxidative damage in crustaceans [63,64]. Cu exposure also induced the expression of *CYP450* (CL1886.Contig2_All) and *CXE2* (CL426.Contig5_All). The aforementioned findings suggest that the prawn *M. rosenbergii* attempts to counteract excessive cellular Cu uptake by initiating detoxification mechanisms in the hepatopancreas.

Accumulation of Cu in the organs of crustaceans leads to the overproduction of ROS, resulting in oxidative damage [10]. NADPH oxidase (NOX) is the main source of ROS in living organisms [65,66]. It has been confirmed that NOX was activated and excessive ROS were produced after Cu exposure in *P. clarkii* [67]. Activation of the antioxidant enzyme system can mitigate the excess ROS [68]. Glutathione peroxidase (GPx) functions in catalyzing the reduction of hydrogen peroxide and lipid peroxide [69]. Glutathione-S-transferase (GST) is involved in cellular antioxidant defense by catalyzing the binding of glutathione to electrophiles [70]. GPx and GST are usually used as indicators of oxidative stress after metal exposure. Moreover, it has been found that up-regulation of *GST* can enhance an organism’s resistance to Cu exposure [71]. However, the present study revealed that *NOX* (CL1542.Contig3_All) was significantly induced at both 3 and 48 h time points, whereas *GST* (CL355.Contig7_All) was suppressed. In addition, the transcription level of glutathione peroxidase (*GPx*) (CL5985.Contig3_All) was up-regulated at 3 h and down-regulated at 48 h. Similar to our results, in *Thamnaconus septentrionalis* exposed to cadmium (Cd), the increase in antioxidant enzyme activity in the short term can effectively remove ROS, but long-term Cd exposure weakens the organism’s antioxidant defense ability [72]. Therefore, we speculated that after Cu exposure, *GPx* has a stronger protective effect on *M. rosenbergii* compared to *GST*. Initially, *GPx* was up-regulated to exert its antioxidant function, but as the duration of exposure prolonged, *GPx* became insufficient to eliminate the sustained excess ROS production, leading to the inhibition of *GPx* expression. Consequently, the accumulation of Cu in the hepatopancreas of *M. rosenbergii* may elevate the risk of oxidative damage.

Oxidative stress caused by ROS can attack biological macromolecules in cells, causing DNA damage and impaired protein function [73]. Previous studies have shown that the heavy metal Cd can inhibit the DNA damage repair mechanism of aquatic organisms [74,75,76]. Similar results were also found in our study. Mismatch repair (MMR) can repair the base mismatch during DNA replication [77]. Nucleotide excision repair (NER) can remove DNA damage caused by various exogenous pressures [78]. After Cu exposure, MutL Homolog 1 (*MLH1*) (Unigene25307_All), MutS Homolog 2 (*MSH2*) (Unigene8072_All), and MutS Homolog 6 (*MSH6*) (Unigene15503_All) in the MMR pathway and DNA damage-binding protein 1 (*DDB1*) (CL4136.Contig1_All), DNA damage-binding protein 2 (*DDB2*) (Unigene362_All), replication factor C (*RFC*) (Unigene27679_All, Unigene768_All, and CL6139.Contig1_All), and DNA polymerase epsilon subunit 2 (*POLE2*) (Unigene17725_All) in the NER pathway were significantly down-regulated. These results indicated that Cu may have a similar genotoxic mechanism to Cd, aggravating cell damage by inhibiting genes related to oxidative damage repair. Heat shock proteins (HSPs) are a highly conserved protein family, which can maintain cellular protein homeostasis against environmental stress and play an important role in cellular stress response [79]. HSP70 and HSP90 are important members of the heat shock protein family, which are responsible for assisting the correct folding, repair, and degradation of proteins, as well as stabilizing key proteins and promoting signal transduction [80]. They are essential for cell survival and the maintenance of normal functions. In our study, Cu exposure inhibited the expression of *HSP70* (CL1286.Contig5_All) and *HSP90* (CL1294.Contig2_All). Previous studies have shown that Cu inhibits the expression of *HSP40* in *Tigriopus japonicus* to mediate cell damage [73]. Similarly, decreased expression levels of *HSP70* and *HSP90* were observed in *M. japonicus* exposed to Cu or Cd [81]. Therefore, we speculated that when *M.rosenbergii* is exposed to a Cu-contaminated environment, the DNA damage repair mechanism and cellular stress response system may be inhibited, thereby further exacerbating oxidative damage.

Apoptosis represents a fundamental physiological and biochemical process, serving as a critical mechanism for the elimination of damaged or mutated cells within the host [82]. Cytochrome c (Cyt C) acts as a pivotal initiating factor in the mitochondrial pathway in cell apoptosis [83]. The forkhead box protein O (FOXO) primarily induces and modulates the expression of the pro-apoptotic protein Bim, thereby enabling cytochrome c (Cyt C) to cross the mitochondrial membrane and activate members of the caspase family, such as caspase 2 (Casp 2), to promote the occurrence of apoptosis [84,85,86]. Survivin, a member of the inhibitor of apoptosis protein (IAP) family, has been demonstrated to decrease the inhibitory action against caspases when its expression is decreased, thus promoting the execution of the apoptotic cascade [87]. In the present study, the expression levels of *Cyt C* (CL4973.Contig1_All), *FOXO* (CL1823.Contig6_All), and *Casp2* (CL1421.Contig1_All) were significantly up-regulated under Cu exposure, while the expression level of *survivin* (CL7764.Contig1_All) was inhibited. These gene expression changes indicated that apoptosis may occur in the hepatopancreatic cells of *M. rosenbergii* during Cu exposure. Previous studies have shown that excessive Cu leads to DNA damage, and if such damage remains unrepaired, cells may undergo a series of complex enzymatically catalyzed reactions resulting in apoptosis [88,89]. Based on these findings, we speculated that the up-regulation of apoptosis-related genes observed in this study may be attributed to the inhibition of the DNA repair system and cellular stress response by Cu, resulting in the organism initiating apoptosis to eliminate damaged cells.

Heavy metal stress could increase the energy demand of crustaceans to cope with metal detoxification and damage repair [90,91]. AMP-activated protein kinase (AMPK) is an energy sensor. When environmental stress causes changes in the energy state of organisms, such as the increase of AMP/ATP ratio, AMPK will be phosphorylated to promote glycolysis and fatty acid oxidation, so as to restore the energy homeostasis in the body [92]. Serine/threonine–protein phosphatase 6 regulatory subunit 3 (SAPS3) is a negative regulator of AMPK, and when metabolic disorders occur, *SAPS3* is inhibited, leading to AMPK phosphorylation [93]. We observed that the expression of *SAPS3* (CL281.Contig5_All) was significantly suppressed after Cu exposure. Moreover, the transcription levels of genes related to fatty acid synthesis, including fatty acid synthase (*FAS*) (Unigene17066_All), acetyl-CoA carboxylase (*ACC*) (CL441.Contig3_All), and delta-9 acyl-CoA desaturase (*SCD1*) (CL800.Contig2_All), were also down-regulated. Glucose transporter 2 (Glut 2) participates in the intracellular uptake and transport of glucose, with an increase in its expression potentially enhancing glucose intake by cells and facilitating glycolysis processes [94,95]. Based on our transcriptome analysis, Cu exposure induced the expression of *Glut 2* (CL1337.Contig2_All). The results presented herein suggest that under Cu exposure, *M. rosenbergii* might potentially enhance its resistance to Cu toxicity by promoting glycolysis and fatty acid oxidation and inhibiting fatty acid synthesis to accommodate the increased energy demand of the organism. 

SSRs and SNPs are essential molecular markers in the fields of genetic diversity analysis and molecular-assisted breeding, among others [96,97]. In recent years, they have been applied to the study of genetic variation in numerous crustacean species [98,99,100]. However, there have been no reports of SSRs or SNPs identified in the hepatopancreas of *M. rosenbergi* under Cu exposure. We identified a significant number of SSRs and SNPs within 85,984 unigenes in the present study. These markers will provide an invaluable genetic resource, facilitating the development of selective breeding programs aimed at enhancing stress resistance in *Macrobrachium* species.

## 5. Conclusions

In this study, transcriptomic analysis was conducted to explore the molecular response mechanisms of the hepatopancreas in *M. rosenbergii* under Cu exposure. The findings indicated that *M. rosenbergii* attempts to resist the toxicity of Cu by up-regulating the expression of genes related to immunity, metabolism, and detoxification. However, with the excessive accumulation of ROS, the antioxidant enzyme system was destroyed. As a result, DNA damage repair and the cellular stress response were inhibited, thereby exacerbating cell damage. In order to maintain the normal function of the hepatopancreas, *M. rosenbergii* removed the damaged cells by activating the apoptosis mechanism. Overall, our study not only facilitates an understanding of the molecular response mechanisms of *M. rosenbergii* underlying Cu toxicity effects but also helps in identifying potential biomarkers associated with the stress response in other crustaceans.

## Figures and Tables

**Table 1:** Primers for qRT-PCR.

Gene Name	Gene ID	Sequence (5′-3′)	Product Length (bp)	Tm (°C)
*Relish*	CL2513.Contig3_All	F: AGGAGGAGGAAGAGGAAGAGGAGAG	164	61
		R: TGGCACTGAAGGCTCATCTGGAA		61
*Glut 2*	CL1337.Contig2_All	F: TGGCTATTTCTCTGGCGGCATTG	120	60
		R: CGATGGAGGCGGAAGTGATGAAC		60
*CXE2*	CL426.Contig5_All	F: ATTGTCAGAGGCAAATGGCAGTGT	141	59
		R: CGAAGACTTCCAGTTGGTGGTTCT		59
*HC*	CL2.Contig14_All	F: GGATGCTCTTGCCCAAGGGTAAG	116	60
		R: AGTGCCGCCGTGCTCTGATT		60
*GST*	CL355.Contig7_All	F: CTTGAAGACCCGAGCCACTATTGAT	166	58
		R: GAAACCATTGAGCCATCCCAAAGC		58
*SAPS 3*	CL281.Contig5_All	F: AGAATGGATACATCTTGGGCTCCCT	128	59
		R: GGTATGGCATCACAGGCAGCAAT		59
*CTL*	CL3394.Contig2_All	F: AGGGCTACTGGATCTGGGTGGA	138	60
		R: TCCGAGGGTAACTTTCTTCCGAGT		60
*EF-1*		F: GAGGAAGATTGAACGCAAGA	152	60
		R: TTAAGGATGCCAGTCTCCAC		60

**Table 2:** Statistics of the sequencing and assembly data.

Summary	0 h	3 h	48 h	Total
Total raw reads	65,384,862	58,978,912	55,185,928	179,549,702
Total clean reads	65,117,218	58,693,126	54,982,780	178,793,124
Total clean read ratio (%)	99.59	99.52	99.63	99.58
Number of unigenes	88,579	99,729	102,062	85,984
Mean length of unigenes (bp)	697.92	735.61	693.59	1077.23
N50 length of unigenes (bp)	1704	1954	1782	2559
N90 length of unigenes (bp)	232	237	226	351

**Table 3:** DEGs related to oxidation–reduction, energy metabolism, apoptosis, copper homeostasis, detoxification, immune response, and DNA damage repair were significantly regulated at 3 h and 48 h of Cu exposure.

Gene ID	Gene	Description	log2FC (3 h)	Regulation	log2FC (48 h)	Regulation
Oxidation–reduction						
CL1542.Contig3_All	*NOX*	NADPH oxidase	3.9906	up	4.2492	up
CL5985.Contig3_All	*GPx*	glutathione peroxidase-like	2.5799	up	−8.2860	down
CL355.Contig7_All	*GST*	glutathione S-transferase	−7.3801	down	−5.0590	down
Energy metabolism						
CL281.Contig5_All	*SAPS 3*	serine/threonine–protein phosphatase 6 regulatory subunit 3	−10.9208	down	−10.6095	down
Unigene17066_All	*FAS*	fatty acid synthase	−1.2389	down	−2.6630	down
CL441.Contig3_All	*ACC*	acetyl-CoA carboxylase	3.0290	up	−3.2607	down
CL800.Contig2_All	*SCD 1*	delta-9 acyl-CoA desaturase	−1.0850	down	−1.8534	down
CL1337.Contig2_All	*Glut 2*	glucose transporter 2	7.4867	up	6.0060	up
Apoptosis						
CL4973.Contig1_All	*Cyt C*	cytochrome c	-	-	2.7752	up
CL1823.Contig6_All	*FOXO*	forkhead box protein O-like	3.5861	up	2.3504	up
CL7764.Contig1_All	*survivin*	survivin	−2.4683	down	−4.5185	down
CL1421.Contig1_All	*Casp 2*	caspase 2	1.3839	up	1.2932	up
Copper homeostasis						
CL2846.Contig2_All	*Ctr 1*	High-affinity copper uptake protein 1	1.3229	up	1.6525	up
Unigene9795_All	*COX 17*	cytochrome c oxidase copper chaperone	1.5825	up	1.6470	up
Unigene13182_All	*Cu-MT*	copper-induced metallothionein	−1.2944	down	4.4623	up
CL3799.Contig3_All	*ATP 7A*	copper-transporting ATPase 1-like	-	-	4.8592	up
Detoxification						
CL1886.Contig2_All	*CYP450*	cytochrome P450	-	-	5.7927	up
CL426.Contig5_All	*CXE 2*	JHE-like carboxylesterase 2	4.3916	up	8.2016	up
Immune response						
CL2.Contig14_All	*HC*	hemocyanin	7.0572	up	7.0599	up
CL3394.Contig2_All	*CTL*	C-type lectin	5.3396	up	3.9633	up
CL4909.Contig1_All	*FLP 2*	ficolin-like protein 2	1.7688	up	2.0525	up
CL5288.Contig1_All	*Domeless*	domeless	2.1386	up	2.1734	up
CL607.Contig1_All	*Stat*	signal transducer and activator of transcription	4.3341	up	1.5504	up
CL4523.Contig2_All	*TLR*	Toll-like receptor	3.8945	up	3.0839	up
CL2513.Contig3_All	*Relish*	relish	10.3394	up	9.2218	up
DNA damage repair						
CL4136.Contig1_All	*DDB1*	DNA damage-binding protein 1	−1.1834	down	−1.3557	down
Unigene25307_All	*MLH1*	MutL Homolog 1	-	-	−4.1634	down
Unigene8072_All	*MSH2*	MutS Homolog 2	-	-	−2.0912	down
Unigene15503_All	*MSH6*	MutS Homolog 6	-	-	−1.8564	down
Unigene362_All	*DDB2*	DNA damage-binding protein 2	-	-	−1.8626	down
Unigene27679_All	*RFC2*	replication factor C subunit 2-like	-	-	−2.1294	down
Unigene768_All	*RFC4*	replication factor C subunit 4	-	-	−2.1227	down
CL6139.Contig1_All	*RFC5*	replication factor C subunit 5-like	-	-	−2.5631	down
Unigene17725_All	*POLE2*	DNA polymerase epsilon subunit 2	-	-	−3.4375	down

## Data Availability

The authors declare that all data supporting the conclusions of this study are available within the article.

## Supplementary Materials

The following supporting information can be downloaded at: https://www.mdpi.com/article/10.3390/ani14071117/s1, Figure S1: Length distribution of all assembled unigenes; Figure S2: Number of unigenes annotated to different databases; Figure S3: Venn diagram of the transcriptomes against the Nr, Nt, COG, SwissProt, and KEGG databases; Figure S4: Distribution of the top 30 species with the most homologous sequences; Figure S5: GO classification of the assembled unigenes; Figure S6: KEGG annotation analysis of the unigenes; Figure S7: Numbers of DEGs in each comparison; Table S1: De novo assembly sequences; Table S2: Annotation results of unigenes in Nr, NT, COG, SwissProt, and KEGG; Table S3: Differentially expressed genes of three comparison groups; Table S4: SSR results and corresponding primers.
