# Supplementary figures and images for: Comparative Transcriptome Analysis of the Hepatopancreas from Macrobrachium rosenbergii Exposed to the Heavy Metal Copper
Source: Animals (Basel). 2024 Apr 5;14(7):1117. doi: 10.3390/ani14071117 (PMC11011146; doi:10.3390/ani14071117)

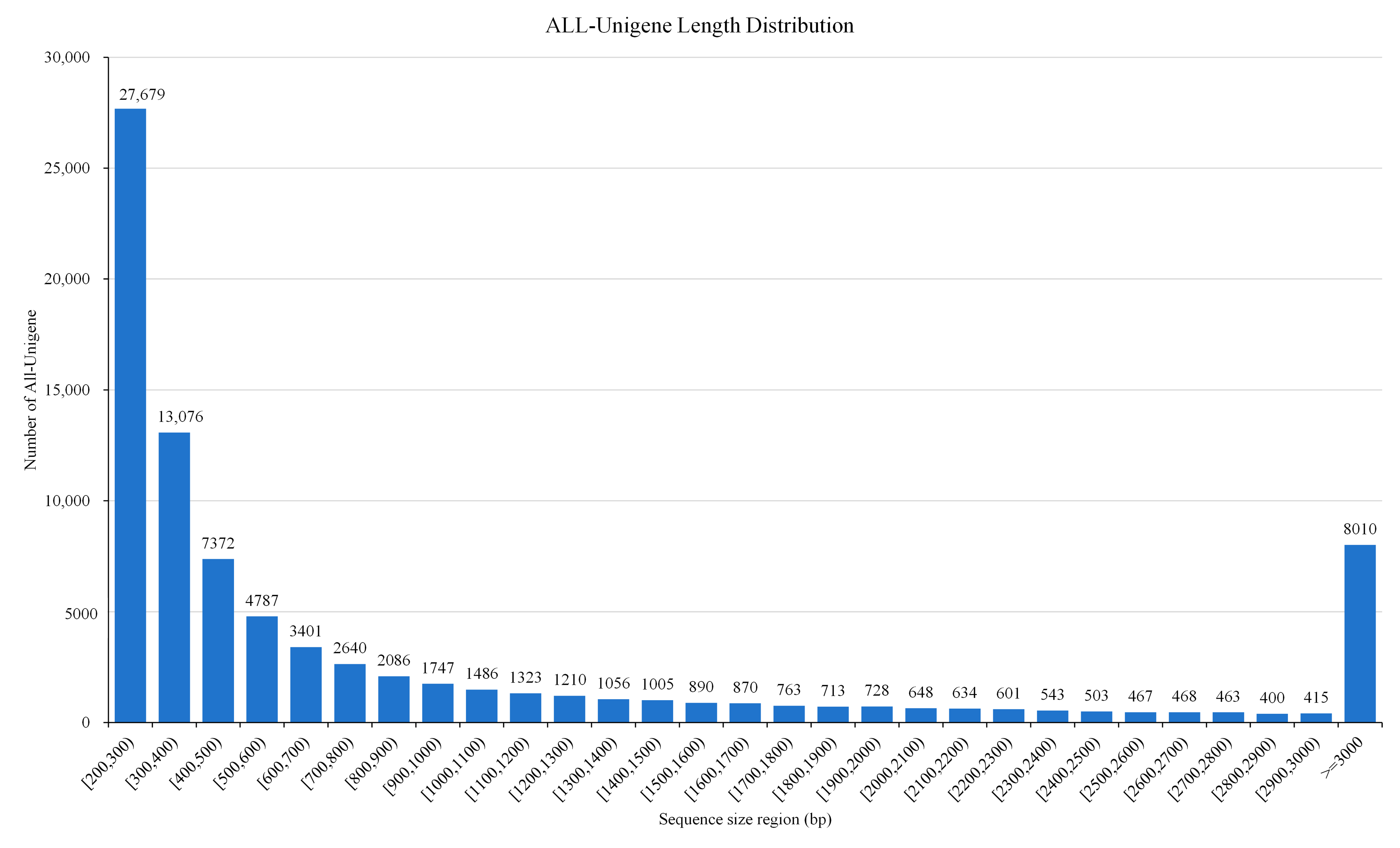

Supplement: Supplementary file 1 [file animals-14-01117-s001.zip › Supplementary Materials-Figure/Figure S1.tif]

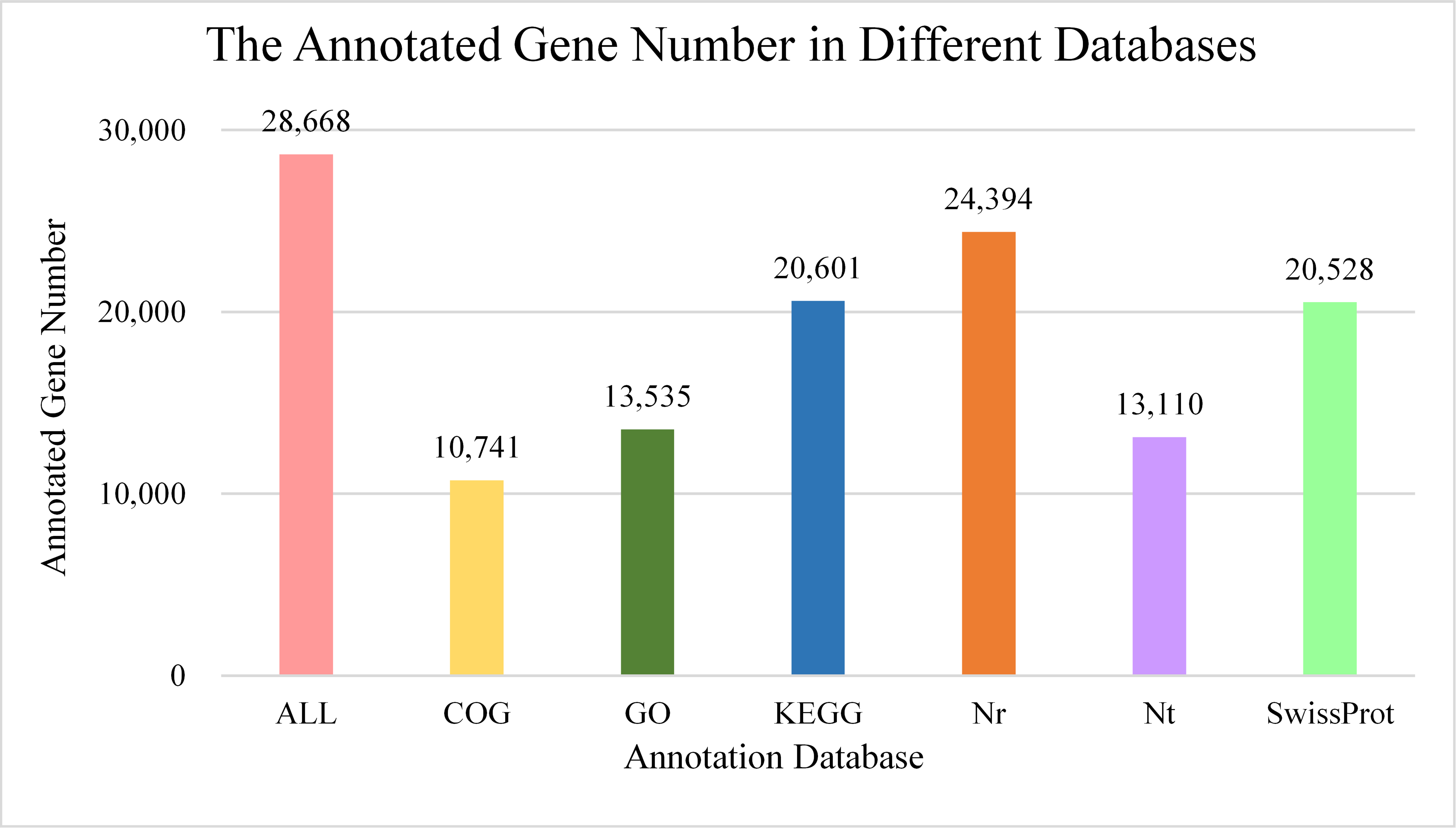

Supplement: Supplementary file 1 [file animals-14-01117-s001.zip › Supplementary Materials-Figure/Figure S2.tif]

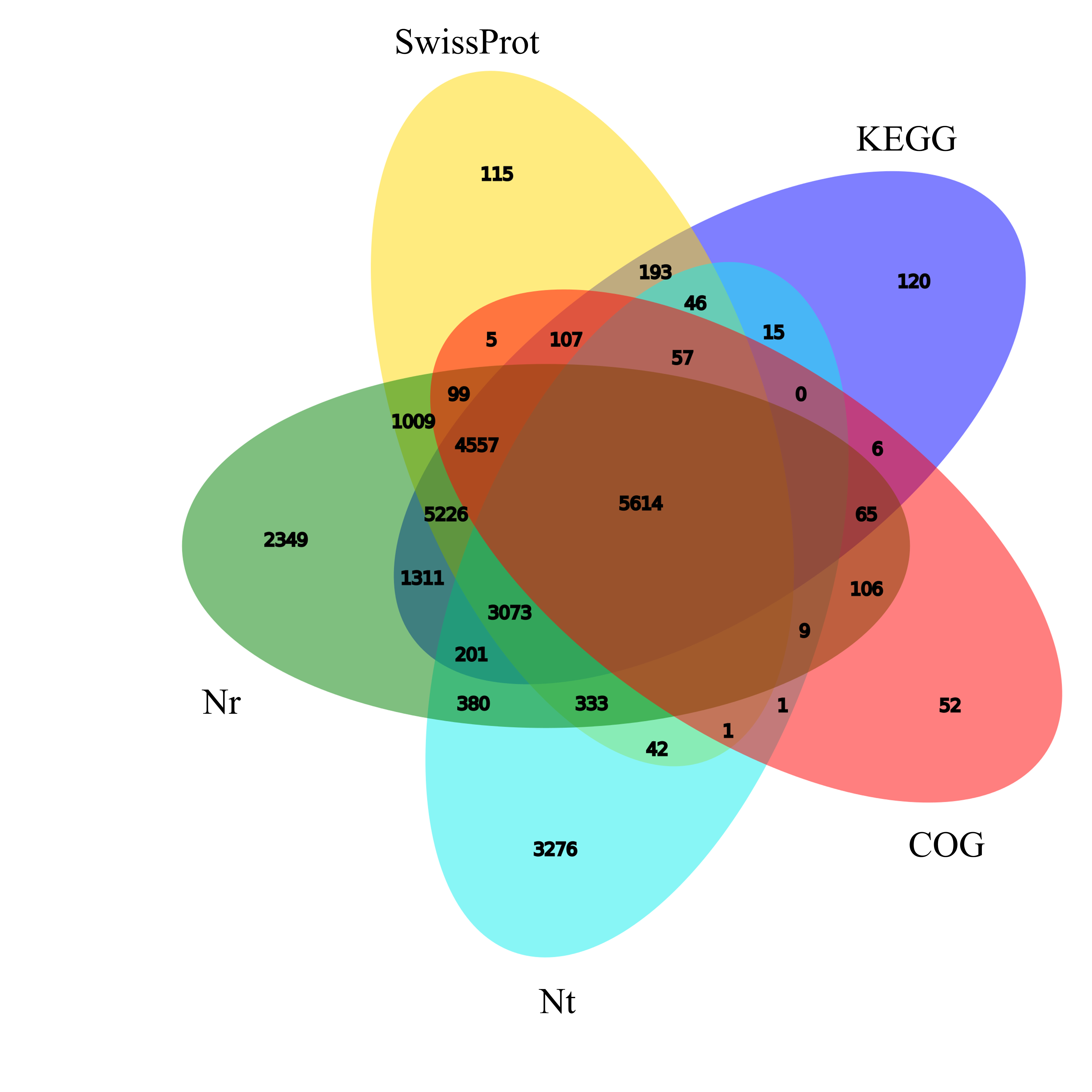

Supplement: Supplementary file 1 [file animals-14-01117-s001.zip › Supplementary Materials-Figure/Figure S3.tif]

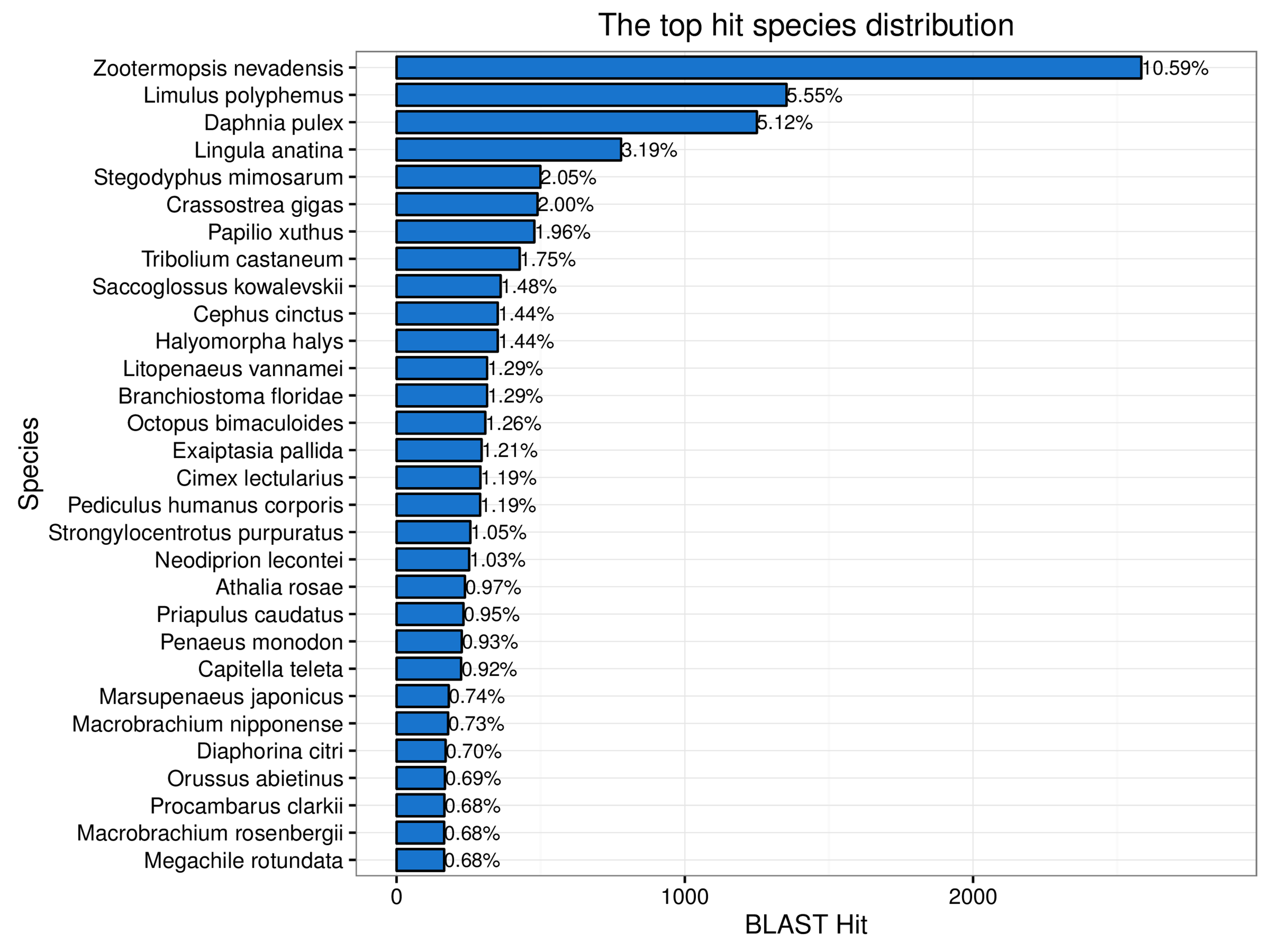

Supplement: Supplementary file 1 [file animals-14-01117-s001.zip › Supplementary Materials-Figure/Figure S4.tif]

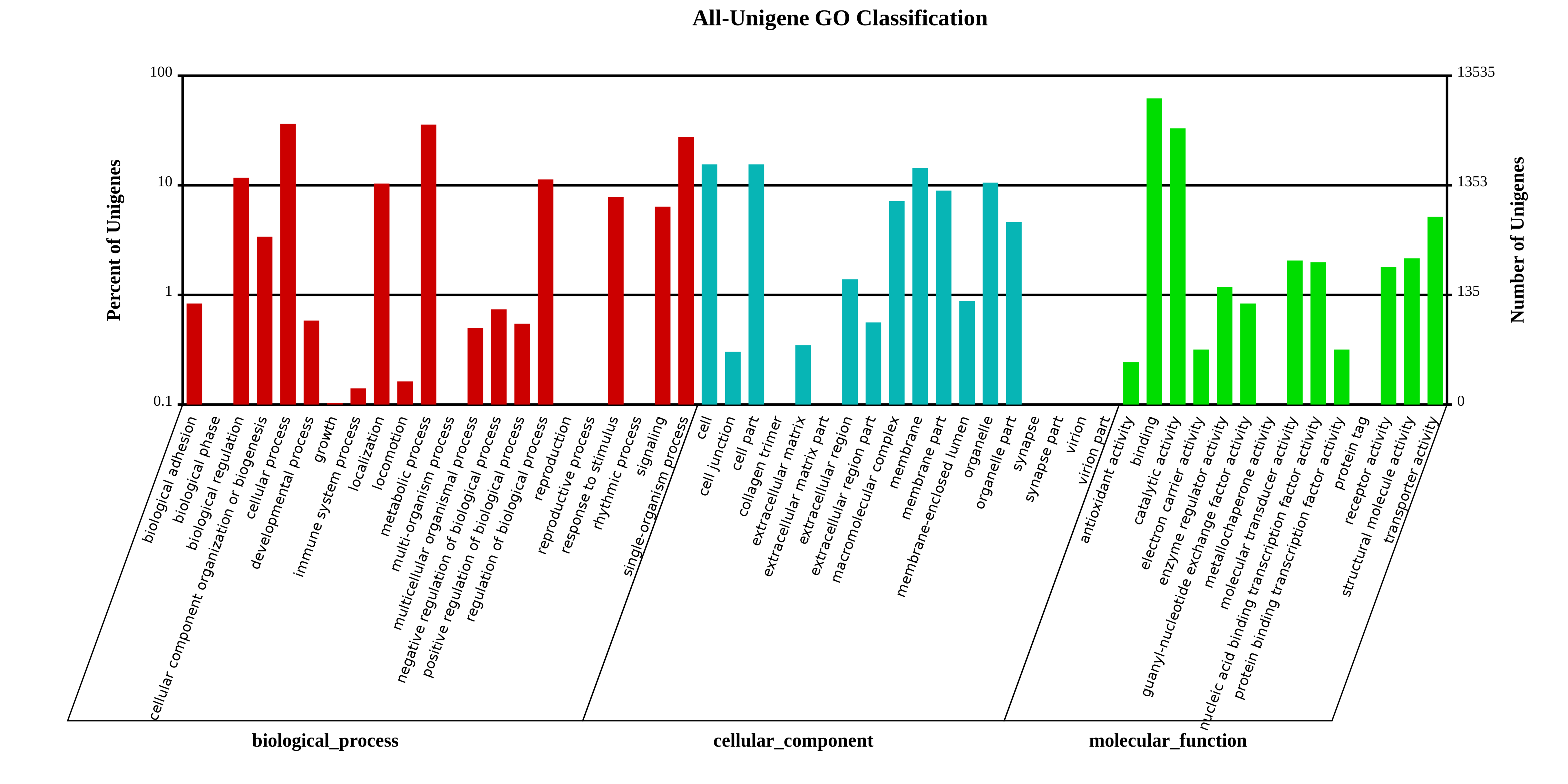

Supplement: Supplementary file 1 [file animals-14-01117-s001.zip › Supplementary Materials-Figure/Figure S5.tif]

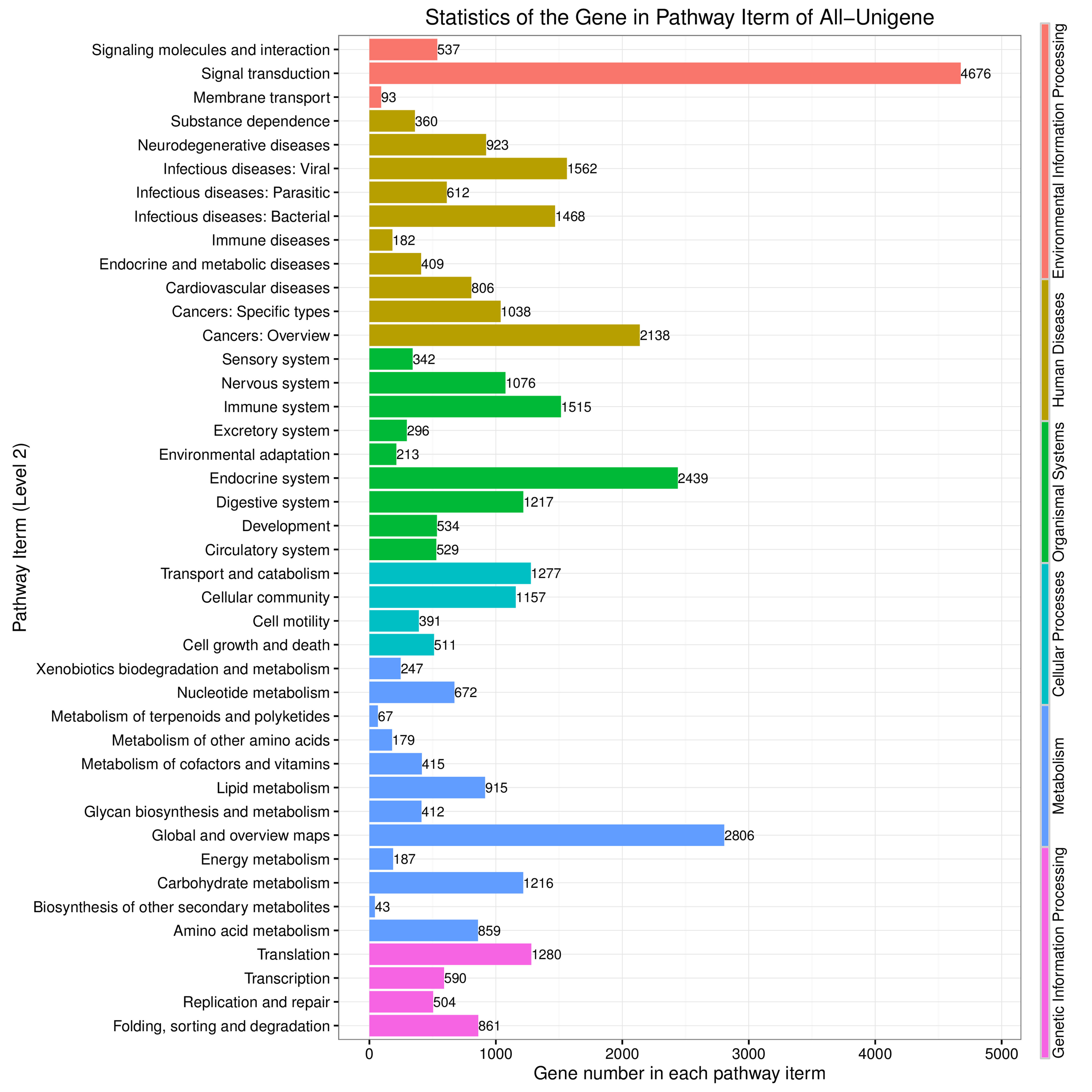

Supplement: Supplementary file 1 [file animals-14-01117-s001.zip › Supplementary Materials-Figure/Figure S6.tif]

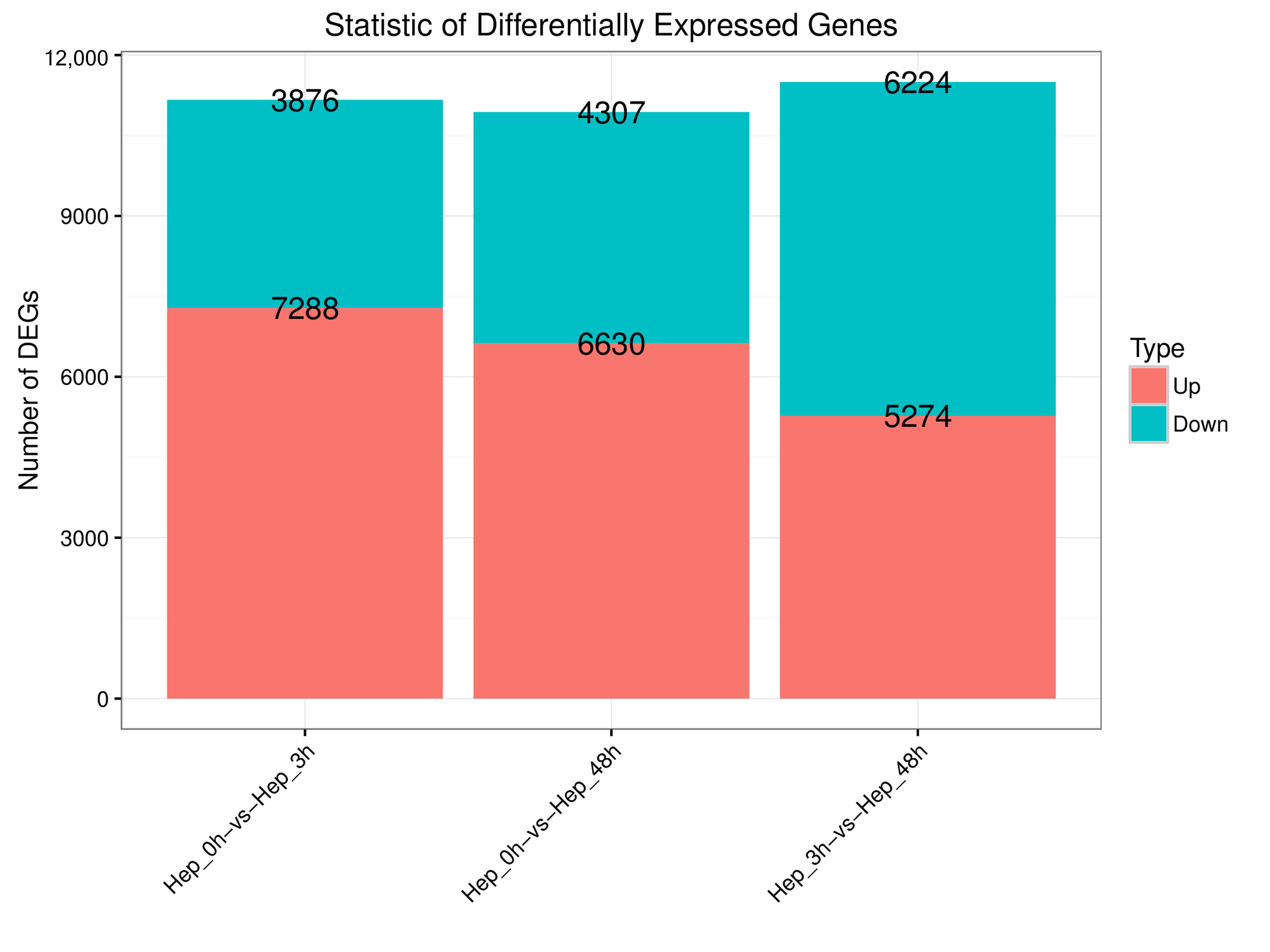

Supplement: Supplementary file 1 [file animals-14-01117-s001.zip › Supplementary Materials-Figure/Figure S7.tif]
